# An Objective Study of Anatomic Shifts in Intracranial Hypotension Using Four Anatomic Planes

**DOI:** 10.1155/2018/6862739

**Published:** 2018-03-11

**Authors:** Shamar J. Young, Ronald G. Quisling, Sharatchandra Bidari, Tina S. Sanghvi

**Affiliations:** ^1^Department of Radiology, University of Minnesota, 420 Delaware St SE, Minneapolis, MN 55455, USA; ^2^Department of Diagnostic Radiology, University of Florida, 1600 SW Archer Rd, Gainesville, FL 32610, USA

## Abstract

**Purpose:**

Intracranial hypotension (IH) often remains undetected using current MR diagnostic criteria. This project aims to demonstrate that central incisural herniation is highly effective in helping to make this diagnosis.

**Materials and Methods:**

Magnetic resonance imaging (MRI) was analyzed in 200 normal and 81 clinically known IH patients. MRI reference lines approximating the plane of the incisura, the plane of the diaphragma sella, the plane of the foramen magnum, and the plane of the visual pathway were utilized to measure the position of selected brain structures relative to these reference lines.

**Results:**

All IH patients had highly statistically significant (*p* < 0.0001) measurable evidence of downward central incisural herniation when compared to normal controls. The first of the important observations was a downward shift of the mammillary bodies, which shortened the midsagittal width of the interpeduncular fossa cistern. A concurrent downward shift and deformity of the tuber cinereum accompanied the mammillary body shift. The second essential observation was an abnormal clockwise rotation of the long axis of the visual pathway. A severity grading system is proposed based on the extent of these shifts as well as secondary shifts of the brain stem, splenium, and cerebellar tonsils.

**Conclusion:**

This study objectively delineates the anatomic shifts of brain structures adjacent to the incisura and foramen magnum. This methodology is sufficient to recognize the features of IH and to stratify the spectrum of IH findings into a functional grading system for quantifying the results of interventional therapy.

## 1. Introduction

While much has been learned about intracranial hypotension (IH) in the last several years, for a variety of reasons, an initial misdiagnosis still remains common [1, 2]. In part, the diagnostic challenge stems from the myriad of reported presenting clinical symptoms. Although the complaint of headache is most common [3], nausea/vomiting [4], cranial neuropathies [5–9], radiculopathies [10], Parkinsonism [11], quadriplegia [12], and even coma [13–17] have also been described. To complicate matters further, the originally described diagnostic finding of low opening cerebral spinal fluid (CSF) pressure has also come into question. Normal CSF pressures have been seen in patients with well documented symptoms of IH, suggesting a wide range for normal baseline CSF pressures as well as significant variation in the pressure decline required to yield symptoms between individuals [18, 19]. Thus CSF volume, rather than the actual CSF pressure, is likely the pathophysiological variable at play [18, 20].

Unfortunately, the clinically experienced diagnostic dilemmas are matched by similar limitations in imaging. Reported magnetic resonance imaging (MRI) features of IH have lacked measureable criteria to provide an objective basis for diagnosis. Similarly, no published criteria exist to grade the extent or severity of IH nor to document interval progression/improvement following treatment. The MRI findings of IH are primarily the result of CSF volume loss and compensatory mechanisms according to the Monroe-Kellie doctrine [5]. Loss of CSF results in loss of sufficient buoyant force to prevent the brain from descending downward. Compensatory subdural effusions can develop, which in some cases also causes rupture of subdural bridging veins transforming subdural hygromas into subdural hematomas and thereby adding additional downward herniation force [19]. In the absence of unilateral subdural hematomas, most cases of IH produce central incisural herniation, rather than the more common asymmetric, or paracentral downward transtentorial herniation typically associated with asymmetric supratentorial mass effect.

Prior reported imaging indicators of IH include subdural fluid collections, pachymeningeal enhancement, engorgement of venous structures, pituitary hyperemia, and “sagging” of the brain [10, 21]. Although these findings may be present in patients with IH, none occur with sufficient frequency to qualify as essential diagnostic criteria. For example, pachymeningeal enhancement is the most well recognized imaging finding of IH, reported in up to 83% of patients [22–30]; however, lack of enhancement does not preclude diagnosis. Messori et al. found subdural fluid collections in less than half of their IH patient population [20]. Brainstem “slumping,” an MRI sign considered specific for IH [28, 31–33], has only been reported in approximately 51% of cases [34]. Thus, in the absence of these reported findings there has been no means of differentiating normal from abnormal in a substantial number of patients. Furthermore, lack of a severity grading scale precludes a means of documenting treatment effectiveness.

The goals for this retrospective review were twofold. This study tests the hypothesis that central incisural herniation is present in IH and can be quantitatively described. This hypothesis is tested by measuring interval distances between relevant brain structures and fixed anatomically based planes of reference both at the tentorial hiatus and at the foramen magnum in both normal and IH patient populations. Secondly, the study aims to stratify IH patients based upon the extent of the brain displacement to create a grading system which can objectively evaluate posttreatment effects.

## 2. Material and Methods

### 2.1. Definition of Planes of Reference and Description of Distances Measured

#### 2.1.1. Description of the Specific Measurements Relative to the Plane of the Incisura Ventral to the Mesencephalon

**Figure 1:**
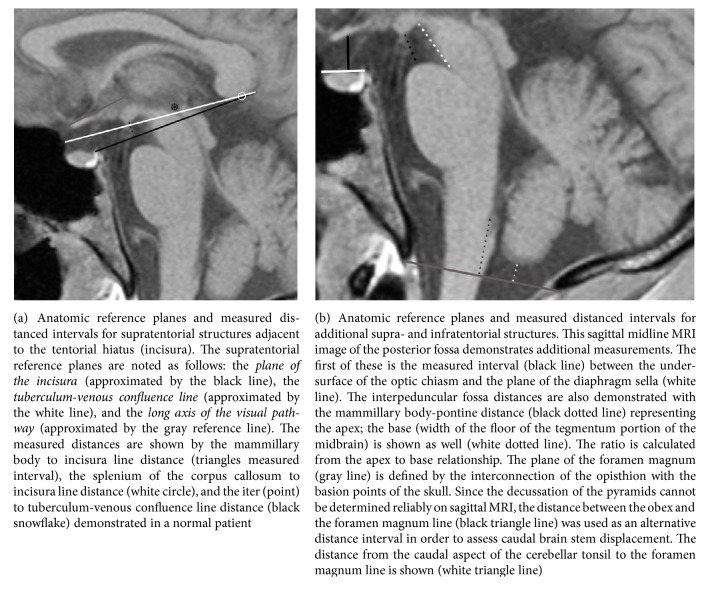
Incisural and foramen magnum related anatomic planes of reference and their MRI equivalent reference lines.* Technical note*. A symbol (rather than a line) has been used in the figures, when a measured distance is close to or equal to zero.

The plane of the incisura, formed by interconnecting the Galenic venous confluence point (which includes the internal cerebral veins, the mesencephalic veins, and the basal veins of Rosenthal) with the base of the dorsum sella (Figure 1), provides the best estimate of the actual tentorial free margins forming the tentorial hiatus. The incisura includes all CSF spaces within the tentorial hiatus as well as the midbrain. Supratentorial structures adjacent to the plane of the incisura but ventral to the midbrain include the tuber cinereum and the mammillary bodies. Apposed structures immediately below the plane of the incisura include the belly of the pons. The interpeduncular fossa crosses the plane of the incisura. Its apex, which is open, is estimated by the distance between the caudal mammillary body surface superiorly and the belly of the pons inferiorly. The base of the interpeduncular fossa is the undersurface of the central midbrain. The normal relationship between these supra- and infratentorial structures and their measurements including the mammillary-pontine distance, height of the interpeduncular cistern at the plane of the cecum, and the ratio of these measurements are illustrated in Figure 1. Objective measurable evidence of downward central incisural herniation is the reduction of the distance between the mammillary bodies and the belly of the pons and the downward displacement of the mammillary bodies toward, then crossing the plane of the incisura. The measurement of the distance from the incisural line to the mammillary bodies is illustrated in Figure 1. Additionally, as central incisural herniation proceeds and the mammillary bodies descend below the plane of the incisura, the normal rectangular configuration of the interpeduncular fossa as seen on the sagittal projection compresses anteriorly forming more of a triangle. This change in interpeduncular shape is an easily discernable finding and is also illustrated in Figure 1.

#### 2.1.2. Description of the Specific Measurements Relative to the Plane of the Incisura Dorsal to the Mesencephalon

The splenium of the corpus callosum is located superior to the plane of the incisura. The distance between the caudal surface of the splenium and the plane of the incisura has been measured and is illustrated in Figure 1.

#### 2.1.3. Description of the Specific Measurements Relative to the Position of the Mesencephalon

The mesencephalon crosses the plane of the incisura with roughly half above and half below. The iter reference point corresponds to the porus of the cerebral aqueduct. The distance between the iter and the plane of the incisura estimates the normal position of the mesencephalon and was measured as illustrated in Figure 1. Reduction in this distance is a measure of downward displacement.

#### 2.1.4. Description of the Specific Measurements Relative to the Plane of the Long Axis of the Visual Pathway

**Figure 2:**
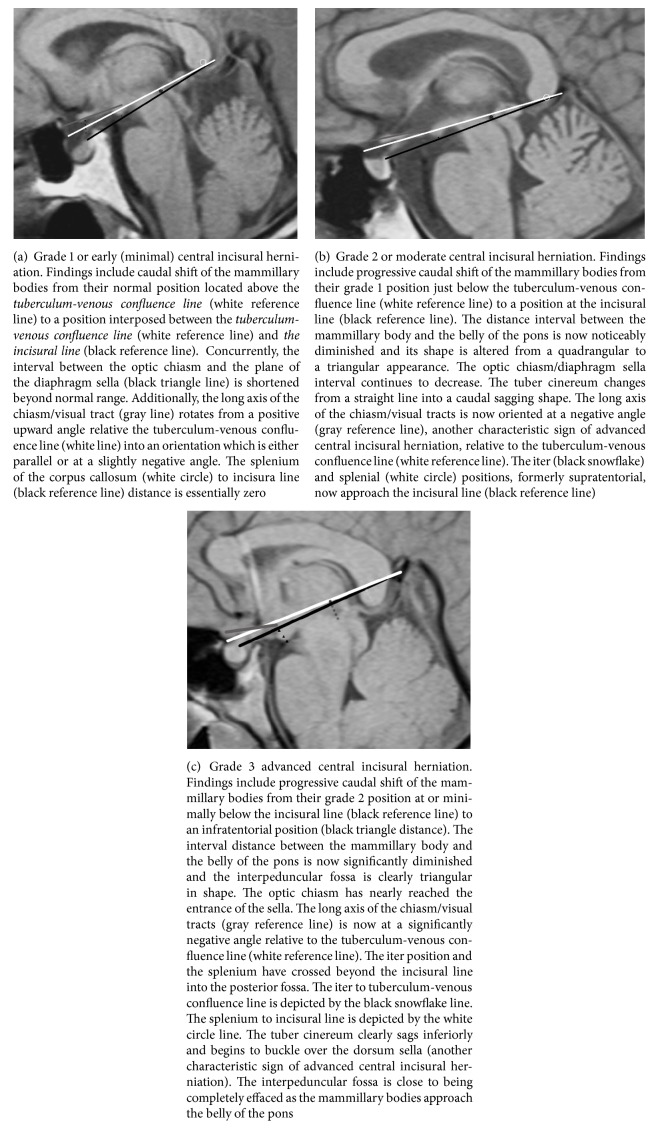
Grading downward central incisural herniation.* Technical note*. A symbol (rather than a line) has been used in the figures when a measured distance is close to or equal to zero.

The second anatomic displacement related to downward central incisural herniation is a clockwise rotation of the visual pathway relative to the plane of the long axis of the visual pathway. The tuberculum-venous confluence reference line provides a means of evaluating the spatial orientation of the visual pathway relative to the central skull base as delineated by the plane of the diaphragm sella. The line is formed by the interconnection between the same Galenic venous confluence mentioned in creation of the plane of the incisura and the tuberculum sella. This is an artificial reference line rather than an anatomic line, but does parallel the normal ascending oblique orientation of the optic chiasm, as shown in Figure 2. This plane of reference can then be used to detect the clockwise rotation of the visual pathway axis in the context of central incisural herniation. This angle was measured and is illustrated in Figure 1.

#### 2.1.5. Description of the Specific Measurements of the Optic Chiasm Position Relative to the Plane of the Diaphragm Sella

The third anatomic displacement related to downward central incisural herniation is downward displacement of the optic chiasm relative to the entrance of the sella that is estimated by the plane of the tuberculum sella, the plane that establishes the position of the diaphragm sella and also delineates the intracranial surface of the central skull base. This reference line represents the interconnection of the tuberculum sella anteriorly with the diaphragm sella insertion point posteriorly. Note, the tuberculum sella is also the anterior point for diaphragm sella insertion. This distance was measured and is illustrated in Figure 1.

#### 2.1.6. Description of the Specific Measurements Relative to the Plane of the Foramen Magnum

The plane of the foramen magnum is created by interconnecting the basion with the opisthion points of the osseous foramen magnum. The decussation of the pyramids, which defines the cervicomedullary junction, is estimated by drawing a vertical line across the medulla from the obex of the caudal fourth ventricle. This methodology has been shown to fall within a few millimeters of the pyramidal decussation [35]. The position of the normal cervicomedullary junction is defined by the distance between the cervicomedullary junction and the plane of the foramen magnum. The position of the caudal poles of the tonsils is defined by the interval between the tonsils and the plane of the foramen magnum. Both the distance of the plane of the foramen magnum to the caudal tip of the cerebellar tonsil and the distance from the plane of the foramen magnum to the obex were obtained as illustrated in Figure 1. These anatomic relationships between the cervicomedullary junction and the cerebellar tonsils relative to the plane of the foramen magnum allow independent assessment of secondary displacement of the brainstem versus the caudal poles of the cerebellar tonsils in the context of central incisural herniation. These changes can sometimes be confused with Chiari malformations.

#### 2.1.7. Additional Imaging Features Associated with IH

All patients, including both normal and IH categories, were evaluated for a presence of the venous engorgement, dural venous sinus distension (as positive if the borders of the dominant transverse sinus were convex rather than concave), presence of subdural hygromas/hematomas, and pituitary enlargement.

### 2.2. Study Description

After institutional review board approval was obtained, a retrospective review of the electronic medical records (EMR) and picture archiving and communication system (PACS) was performed of patients undergoing brain MRIs from 3/2004 to 9/2012. Inclusion into the IH group required clinical and radiographic evidence of IH that met the international classification of headache disorders 3rd edition criteria for IH. A list was obtained from our clinical colleagues and a search of the radiology report database was performed for “intracranial hypotension.” This produced a group of patients with both clinical and MRI findings of IH. If findings of IH were found on MRI only and the patient was not on the list provided by our clinical colleagues, the EMR was searched to determine whether patients did indeed meet HIS-3 criteria for IH. Those that did were also included. Exclusionary criteria included hydrocephalus, acute global brain swelling, and pseudotumor cerebri as seen on MRI, all of which can also produce central incisural herniation.

The normal and IH patient cohorts were retrospectively reviewed independently by two attending neuroradiologists (RQ and TS). The control group consisted of patients imaged during the same time period without clinical symptomatology of IH and who were felt, after review by the same two neuroradiologists, to have normal brain MRIs. All distance measurements were obtained using midline, T1-weighted, sagittal-projection, MRI sequences using 3 mm slice thicknesses. Measurements were given a positive value when the structure of interest was located above the reference line and a negative number if below. The mean ages of normal and IH patients were compared using a *t*-test. Fishers exact test was used to compare the proportions of males and females in the two groups. All analyses were completed in SAS 9.3. Means and standard deviations for normal (*n* = 200) and all IH patients (*n* = 81) as well as for IH patients with grades 1, 2, and 3 were calculated. For each response variable, the means of normal and IH patients were compared, controlling for sex and the potential interaction in sex and normal/IH patients.

**Figure 3:**
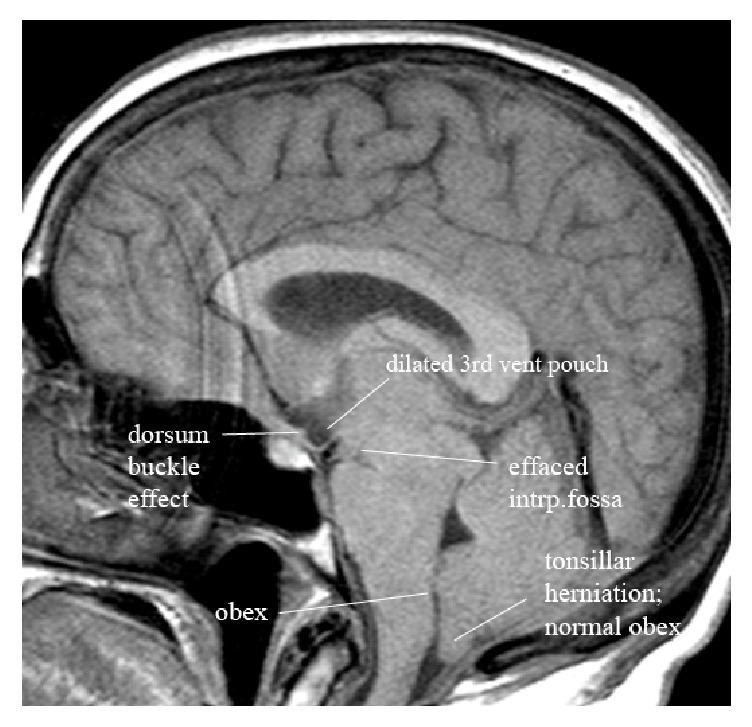
Very advanced grade 3 central incisural herniation. Imaging features of the most advanced state are presented in this figure. Notice that in this most advanced state of central incisural herniation the mammillary bodies have reached the belly of the pons completely effacing the interpeduncular fossa. The tuber cinereum typically buckles over the dorsum sella (a second characteristic sign of central incisural herniation), which creates a pocket of CSF within the lower third ventricle. The suprasellar space is effaced as the optic chiasm position reaches the entrance to the sella. The long axis of the chiasm/visual tracts is oriented at a significantly negative angle relative to the* tuberculum-venous confluence line*. The iter and the splenium have crossed the* incisural line* well into the posterior fossa. The obex position is low and the cerebellar tonsils are also herniated beyond normal range.

A simple grading system for downward central incisural herniation was created by assessing the mammillary bodies' relationship to the incisural line. Grade 1 displacement is defined as mammillary body position above the incisural line. In grade 2 they reach the incisural line and in grade 3 they descend below the incisural line. These grades are illustrated in Figures 2 and 3.

## 3. Results

A total of 81 consecutive patients with IH and 200 normal patients were examined. The mean age of 40.4 years (range 13–81 years) for IH patients was not significantly different than the mean age of 39.9 years (range 0–94 years) for normal patients (*p* > 0.05). The normal group consisted of 45% (90) women and 55% (110) men, which differed significantly from the IH cohort which had 74% (60) women and 26% (21) men (*p* = 0.02).

A total of ten interval measurements were made examining the changes related to the visual pathway, the incisura, and the foramen magnum. For each, the mean and standard deviations for each measurement in both the normative group and the IH groups (including grade 1 (early), grade 2 (moderate), and grade 3 (advanced)) are presented in Table 1. With the exception of the distance from the mammillary body tip to the incisura line the difference in normal and IH groups did not differ significantly for females and males.  Table 2 provides the *p* values for these variables between the normative group and displacements in the IH grades. The* p* values for the distance from the mammillary body tip to the incisura line are also provided in Table 2 for both males and females as the interaction between the sexes was significant. Comparison between all IH patients and the normative group is not included but resulted in *p* values < 0.0001 in all cases.

Although the complete data can be found in Tables 1 and 2, some of the most important results are highlighted here. The normal visual pathway angle is initially positive, 7.42 degrees; however, downward central incisural herniation causes a progressive clockwise radial shift measuring −0.68 degrees (IH grade 1), diminishing to −1.19 degrees (IH grade 2) and finally to −9.71 degrees (IH grade 3). The mammillopontine distance progressively decreases, as evidenced by mean measurements of 7.07 mm (normal), 5.32 mm (grade 1), 4.60 mm (grade 2), and 3.79 mm (grade 3). The mammillary body tip to incisura line also progressively decreases from 3.4 mm (normal) to 2.26 mm (IH grade 1), 0 mm (IH grade 2), and −3.8 mm (IH grade 3).

The presence of the classical findings of IH was also investigated. Subdural hygromas/hematomas were seen in 48%, 41%, and 39% of grade 1, grade 2, and grade 3 IH patients, respectively. Dural thickening/enhancement was seen in 63%, 53%, and 50% of grade 1, grade 2, and grade 3 IH patients, respectively. Pituitary enlargement was seen in 22%, 29%, and 39% of grade 1, grade 2, and grade 3 IH patients, respectively, while venous distension was seen in 26%, 35%, and 36% of grade 1, grade 2, and grade 3 patients, respectively. None of these abnormalities were seen in the normal patient cohort.

## 4. Discussion

IH remains a difficult clinical diagnosis, made more difficult because although CSF pressure measurements often fall objectively into the normal range, they are relatively low in symptomatic patients [18, 19]. Evidence of central incisural downward herniation was evident in every case of symptomatic IH. Thus, understanding the primary structural shifts related to IH versus those which occur secondarily is essential.

The need to provide objective measures for the detection of IH has been addressed by a couple authors previously. Pannullo et al. investigated the position of the cerebellar tonsils relative to the foramen magnum and the position of the iter relative to the incsiural line in 7 patients with IH, comparing them to normative data from Reich et al. [24, 36]. They found that downward displacement of the tonsils and/or iter was present in 6 of 7 patients [24]. Messori et al. described 4 different anatomical measurements (the position of the cerebellar tonsils, fourth ventricle, and infundibular recess as well as the angle between the bicommissural line and a line tangential to the floor of the fourth ventricle) in 8 patients with IH, comparing them to 89 normal controls [20]. A subsequent analysis revealed that the cerebellar tonsils, fourth ventricle, and infundibular recess measurements were statistically different between the IH and normal controls. However, the number of patients with IH reviewed was low (less than 10) in each of these papers and they reviewed less anatomic measurements than this study has.

This paper objectively demonstrates several interesting findings in terms of anatomic pathophysiology in IH patients. Progressive clockwise rotation of the visual (optic tract) axis occurs as the lateral geniculate bodies of the thalami progressively descend downward with increasing severity of IH, as defined by our proposed grading system. Detection of this rotational displacement is achieved by comparing the long axis of the visual pathway to the tuberculum-venous confluence reference line. In abnormal IH patients it switches to a negative radial orientation, an important diagnostic feature of central incisural herniation, especially in early stages of IH when findings can otherwise easily be missed. Additionally and concurrent with rotatory visual pathway shift, there is depression of the optic chiasm relative to the diaphragma sella line. In advanced IH the optic chiasm actually reaches the entrance to the sella.

Central incisural herniation affects the mammillary bodies and the tuber cinereum differently. The downward displacement of the mammillary bodies begins from its normal position above both the tuberculum-venous confluence line and the incisural line. Caudal displacement of the mammillary bodies is stratified into grades of severity based upon which reference lines are crossed. In grade 1 displacement mammillary body position is below the tuberculum-venous confluence line but above the incisural line. In grade 2, the mamillary bodies reach the incisural line and in grade 3 they descend below the incisural line (see Figures 2(a)–2(c)). The mammillopontine distance was also studied by Shah et al. 2013 who found cutoff values of 5.5 mm or less for the mammillopontine distance (and 50° or less for the pontomesencephalic angle) to be both sensitive and specific in strengthening the MRI diagnosis of intracranial hypotension. Our results are very similar as the mammillopontine distance average seen in grade 1 is 5.32 mm with a standard deviation of 1.58 mm (Table 1), with decreasing values as the grade progresses [37].

This study brings to light two additional MRI signs not previously reported: the closure of the interpeduncular fossa and buckling of the tuber cinereum over the dorsum. The deformity of the interpeduncular fossa was measured by calculating a ratio of the decreasing apex width versus the unchanging base width. In practical terms this translates into shift from a rectangular shape of the interpeduncular fossa to a triangular shape (see Figures 2(b) and 2(c)). In the most advanced state of incisural herniation, the interpeduncular fossa is completely effaced (see Figure 3). The caudal sag and buckling of the tuber cinereum are illustrated in Figures 1, 2(b), and 2(c). Recognizable caudal shift of the rostral brain stem was measured by downward shift of the iter relative to the incisural line. Caudal displacement of the splenium was recognized in the same manner. Both were caudally displaced only in the more advanced cases of IH. Again, Shah et al. also found no statistically significant difference in the lateral ventricular angle, also referred to as the “corpus callosal angle,” between control subjects and patients with intracranial hypotension [37].

Evidence of concurrent caudal brain stem displacement was based on evidence that the cervicomedullary junction (using the obex as a landmark) approached or passed the foramen magnum line. The posterior fossa structures were among the most stable in our analysis and thus are not a useful finding in either diagnosis or grading IH severity.

The commonly quoted hallmarks of IH including pituitary engorgement, subdural hygroma/hematoma, pachymeningeal enhancement, and venous distension were distributed sporadically across this data set. When present, these findings are certainly helpful in diagnosing IH. However, they were evident in roughly the same proportion across all three severity grades and therefore fail as a means of stratification. They also were not present with sufficient incidence to be considered an essential IH diagnostic finding. The fact that subdural hygroma/hematoma and pachymeningeal enhancement did not increase in prevalence with increasing grade may suggest that these patients have slowly developed severe IH rather than suffering acute onset or that the variation of compensatory mechanisms and factors leading to the presentation of the secondary factors is greater than previously noted. However, further investigation into this area is needed.

The stratification of the data in the IH group into mild, moderate, and advanced, corresponding to IH grades 1, 2, and 3, was born from a need to evaluate the consequence of IH therapy using objective reproducible criteria. The movement of structures relative to these anatomically based reference planes provides such criteria.

Table 3 gives our recommended values for each measurement which could be considered abnormal; however, upon review of the measurement data and grading system, two key structural relationships are recommended to diagnose IH. When the mammillary bodies descend to or beyond the level of the incisural line, the patient has substantial caudal migration and is considered grade 2 (if at the line) or grade 3 (if below the line). In this setting, no further assessment is necessary to confirm significant central incisural herniation. However, if the patient's mammillary bodies remain above the incisural line but below the tuberculum-venous confluence line and the patient has symptoms compatible with IH measuring the angle of the visual pathway is the next step. If the long axis visual pathway angle is more than 1 positive degree compared to the tuberculum-venous confluence line, it falls within normal limits. If the visual angle parallels or is less (i.e., negative angle), then findings are indicative of early IH-related central incisural herniation and proactive spinal imaging or testing may be considered.

This study has several limitations, including its retrospective nature. The grading system which is introduced primarily to provide an objective method of following posttreatment changes is not correlated with patient symptoms, an area of anticipated future research for this group. The grading system will also need validation. The reproducibility of the lines was also not tested directly in this study. The lines were utilized by multiple members of the research group; however differences were not scientifically addressed and this is yet another area of interest for further investigation by this group. Another weakness is the inability to reliably correlate MRI findings with onset of symptoms, as some believe that symptoms and MRI findings may become more profound over time. Lastly while it is the practice at our institution to image all patients with suspected IH by MRI given the IHS-III diagnostic clinical guidelines, this study may have missed potential IH patients. In particular, those patients with very mild symptoms and those not meeting HIS-III criteria may have been missed.

In conclusion, this study reveals that IH is always accompanied by central incisural herniation and changes to the visual pathway. Supratentorial structures ventral to the mesencephalon are most susceptible to central incisural shift. The study of this movement allowed a grading system to be suggested, allowing patients to be objectively followed after therapy. We also suggest an objective means for diagnosing subtle cases of IH. Intracranial changes previously reported in the literature, especially associated with venous congestion in the pituitary gland and dura, were not consistent findings and therefore are considered adjunctive findings, although helpful when present.

## Figures and Tables

**Table 1:** Average measurements of recorded distances, comparing normal with grades of IH.

Variable	Normal	All IH	Grade 1	Grade 2	Grade 3
Distance from mammillary body tip to incisura line	3.39 (1.90)	−0.98 (3.47)	2.26 (1.19)	0 (0)	−3.80 (3.05)

Distance from the diaphragm sella to the optic chiasm	5.16 (1.60)	2.07 (1.62)	3.53 (1.37)	1.8 (1.19)	1.12 (1.12)

Angle of the optic chiasm	7.42^*∗*^ (3.93)	−4.95^*∗*^ (10.2)	−0.68^*∗*^ (7.63)	−1.19^*∗*^ (5.41)	−9.71^*∗*^ (11.5)

Height of the interpeduncular cistern at the plane of the cecum	12.76 (1.84)	11.03 (2.24)	11.61 (1.79)	11.51 (1.67)	10.39 (2.61)

Mammillopontine distance	7.07 (1.33)	4.47 (1.70)	5.32 (1.58)	4.6 (1.53)	3.79 (1.61)

Ratio of interpeduncular cistern	0.56 (0.11)	0.40 (0.14)	0.46 (0.11)	0.40 (0.12)	0.36 (0.15)

Distance from iter to tuberculum-venous confluence line	0.20 (1.93)	−2.51 (4.28)	−0.62 (2.78)	−1.16 (3.12)	−4.67 (4.82)

Distance from splenium of the corpus callosum to the incisura line	0.99 (1.57)	−0.73 (2.72)	0.52 (1.91)	0.06 (1.83)	−2.01 (3.02)

Distance from caudal pole of tonsils to plane of the foramen magnum	1.98 (2.45)	−2.20 (5.80)	−3.31 (7.88)	−1.55 (3.48)	−1.71 (4.91)

Distance from obex to plane of foramen magnum	8.36 (2.95)	5.30 (4.4)	4.98 (5.73)	5.27 (3.48)	5.52 (3.93)

All measurements without *∗* are in mm with standard deviations provided in (). *∗* demarks measurements in degrees.

**Table 2:** *p* values of Table 2 measurements.

*Variable*	Nrl-1	Nrl-2	Nrl-3	1-2	1–3	2-3
Distance from mammillary body tip to incisura line	0.04 (0.10)	<0.0001 (<0.0001)	<0.0001 (<0.0001)	0.05 (0.002)	<0.0001 (<0.0001)	<0.0001 (<0.0001)

Distance from the diaphragm sella to the optic chiasm	<0.0001	<0.0001	<0.0001	0.0007	<0.0001	0.28

Angle of the optic chiasm	<0.0001	<0.0001	<0.0001	0.93	<0.0001	<0.0001

Height of the interpeduncular cistern at the plane of the cecum	0.007	0.02	<0.0001	0.89	0.04	0.10

Mammillopontine distance	<0.0001	<0.0001	<0.0001	0.07	0.0006	0.24

Ratio of interpeduncular cistern	<0.0001	<0.0001	<0.0001	0.08	0.009	0.59

Distance from iter to tuberculum-venous confluence line	0.11	0.11	<0.0001	0.76	<0.0001	<0.0001

Distance from splenium of the corpus callosum to the incisura line	0.32	0.07	<0.0001	0.40	<0.0001	0.0001

Distance from caudal pole of tonsils to plane of the foramen magnum	<0.0001	0.0003	<0.0001	0.23	0.12	0.86

Distance from obex to plane of foramen magnum	<0.0001	0.0008	0.0008	0.96	0.50	0.60

Table providing *p* values. Nrl = normal patient cohort, 1 = grade 1 IH patients, 2 = grade 2 IH patients, and 3 = grade 3 IH patients.

**Table 3:** Suggested cutoff distances for the proposed grades.

Variable	Suggested cutoff values
Distance from mammillary body tip to incisura line	1.25
Distance from the diaphragm sella to the optic chiasm	3.65
Angle of the optic chiasm	1.7^*∗*^
Height of the interpeduncular cistern at the plane of the cecum	11.85
Mammillopontine distance	5.75
Ratio of interpeduncular cistern	0.49
Distance from iter to tuberculum-venous confluence line	−1.15
Distance from splenium of the corpus callosum to the incisura line	0.2
Distance from caudal pole of tonsils to plane of the foramen magnum	−0.1
Distance from obex to plane of foramen magnum	6.85

All suggested values are in mm except for those denoted by *∗* which are in degrees.

## References

[1] Hoseman G. (1909). cited by Bell WE. *Nachwirkengen der Lumbalanasthesie und ihre Benampfung. Verhandl. deutsch. path. Gesellsch. Chir*.

[2] Schievink W. I. (2003). Misdiagnosis of spontaneous intracranial hypotension. *JAMA Neurology*.

[3] Schievink W. I., Morreale V. M., Atkinson J. L. D., Meyer F. B., Piepgras D. G., Ebersold M. J. (1998). Surgical treatment of spontaneous spinal cerebrospinal fluid leaks. *Journal of Neurosurgery*.

[4] Zwicker J., Lum C. (2009). A treatable mimic of Chiari malformation with syringomyelia. *The Canadian Journal of Neurological Sciences Le Journal Canadien Des Sciences Neurologiques*.

[5] Berlit P., Berg-Dammer E., Kuehne D. (1994). Abducens nerve palsy in spontaneous intracranial hypotension. *Neurology*.

[6] Horton J. C., Fishman R. A. (1994). Neurovisual findings in the syndrome of spontaneous intracranial hypotension from dural cerebrospinal fluid leak. *Ophthalmology*.

[7] Warner G. T. A. (2002). Spontaneous intracranial hypotension causing a partial third cranial nerve palsy: A novel observation. *Cephalalgia*.

[8] Ferrante E., Savino A., Brioschi A., Marazzi R., Donato MF., Riva M. (1998). Transient oculomotor cranial nerves palsy in spontaneous intracranial hypotension. *J Neurosurg Sci*.

[9] Brady-McCreery K. M., Speidel S., Hussein M. A. W., Coats D. K. (2002). Spontaneous intracranial hypotension with unique strabismus due to third and fourth cranial neuropathies. *Binocular Vision & Strabismus Quarterly*.

[10] Schievink W. I. (2006). Spontaneous spinal cerebrospinal fluid leaks and intracranial hypotension. *Journal of the American Medical Association*.

[11] Pakiam A. S.-Ï., Christine L., Lang A. E. (1999). Intracranial hypotension with parkinsonism, ataxia, and bulbar weakness. *JAMA Neurology*.

[12] Schievink W. I., Maya M. M. (2006). Quadriplegia and cerebellar hemorrhage in spontaneous intracranial hypotension. *Neurology*.

[13] Beck C. E., Rizk N. W., Kiger L. T., Spencer D., Hill L., Adler J. R. (1998). Intracranial hypotension presenting with severe encephalopathy. *Journal of Neurosurgery*.

[14] Evans R. W., Mokri B. (2002). Spontaneous intracranial hypotension resulting in coma. *Headache: The Journal of Head and Face Pain*.

[15] Ferrante E., Arpino I., Citterio A., Savino A. (2009). Coma resulting from spontaneous intracranial hypotension treated with the epidural blood patch in the Trendelenburg position pre-medicated with acetazolamide. *Clinical Neurology and Neurosurgery*.

[16] Kashmere J. L., Jacka M. J., Emery D., Gross D. W. (2004). Reversible coma: A rare presentation of spontaneous intracranial hypotension. *Canadian Journal of Neurological Sciences*.

[17] Kremer S., Taillandier L., Schmitt E. (2005). Atypical clinical presentation of intracranial hypotension: Coma [1]. *Journal of Neurology*.

[18] Mokri B., Hunter S. F., Atkinson J. L. D., Piepgras D. G. (1998). Orthostatic headaches caused by CSF leak but with normal CSF pressures. *Neurology*.

[19] Paldino M., Mogilner A. Y., Tenner M. S. (2003). Intracranial hypotension syndrome: a comprehensive review. *Neurosurgical Focus*.

[20] Messori A., Simonetti B. F., Regnicolo L., Di Bella P., Logullo F., Salvolini U. (2001). Spontaneous intracranial hypotension: The value of brain measurements in diagnosis by MRI. *Neuroradiology*.

[21] Spelle L., Boulin A., Tainturier C., Visot A., Graveleau P., Pierot L. (2001). Neuroimaging features of spontaneous intracranial hypotension. *Neuroradiology*.

[22] Franzini A., Messina G., Nazzi V. (2009). Spontaneous intracranial hypotension syndrome: A novel speculative physiopathological hypothesis and a novel patch method in a series of 28 consecutive patients - Clinical article. *Journal of Neurosurgery*.

[23] Brightbill T. C., Goodwin R. S., Ford R. G. (2000). Magnetic resonance imaging of intracranial hypotension syndrome with pathophysiological correlation. *Headache: The Journal of Head and Face Pain*.

[24] Pannullo S. C., Reich J. B., Krol G., Deck M. D. F., Posner J. B. (1993). MRI changes in intracranial hypotension. *Neurology*.

[25] Spelle L., Boulin A., Pierot L., Graveleau P., Tainturier C. (1997). Spontaneous intracranial hypotension: MRI and radionuclide cisternography findings [5]. *Journal of Neurology, Neurosurgery & Psychiatry*.

[26] Bruera O. C., Bonamico L., Giglio J. A., Sinay V., Leston J. A., Figuerola M. D. L. (2000). Intracranial hypotension: The nonspecific nature of MRI findings. *Headache: The Journal of Head and Face Pain*.

[27] Mittl R. L., Yousem D. M. (1994). Frequency of unexplained meningeal enhancement in the brain after lumbar puncture. *American Journal of Neuroradiology*.

[28] Mokri B., Piepgras D. G., Miller G. M. (1997). Syndrome of orthostatic headaches and diffuse pachymeningeal gadolinium enhancement. *Mayo Clinic Proceedings*.

[29] Schoffer K. L., Benstead T. J., Grant I. (2002). Spontaneous intracranial hypotension in the absence of magnetic resonance imaging abnormalities. *Canadian Journal of Neurological Sciences*.

[30] Schievink W. I., Tourje J. (2000). Intracranial hypotension without meningeal enhancement on magnetic resonance imaging: case report. *Journal of Neurosurgery*.

[31] Schievink W. I., Meyer F. B., Atkinson J. L. D., Mokri B. (1996). Spontaneous spinal cerebrospinal fluid leaks and intracranial hypotension. *Journal of Neurosurgery*.

[32] Schievink W. I. (2000). Spontaneous spinal cerebrospinal fluid leaks: a review.. *Neurosurgical focus [electronic resource].*.

[33] Fishman R. A., Dillon W. P. (1993). Dural enhancement and cerebral displacement secondary to intracranial hypotension. *Neurology*.

[34] Schievink W. I., Maya M. M., Louy C., Moser F. G., Tourje J. (2008). Diagnostic criteria for spontaneous spinal CFS leaks and intracranial hypotension. *American Journal of Neuroradiology*.

[35] Quisling R. G., Quisling S. G., Parker Mickle J. (1993). Obex/nucleus gracilis position: Its role as a marker for the cervicomedullary junction. *Pediatric Neurosurgery*.

[36] Reich J. B., Sierra J., Deck M. D. F., Plum F. (1993). MRI description and clinical correlation of dynamic upward and downward transtentorial and foramen magnum brain herniation. *Annals of Neurology*.

[37] Shah L. M., McLean L. A., Heilbrun M. E., Salzman K. L. (2013). Intracranial hypotension: Improved MRI detection with diagnostic intracranial angles. *American Journal of Roentgenology*.

